# Stability and Synchronization for Discrete-Time Complex-Valued Neural Networks with Time-Varying Delays

**DOI:** 10.1371/journal.pone.0093838

**Published:** 2014-04-08

**Authors:** Hao Zhang, Xing-yuan Wang, Xiao-hui Lin, Chong-xin Liu

**Affiliations:** 1 Faculty of Electronic Information & Electrical Engineering, Dalian University of Technology, Dalian, China; 2 Institute of Electrical Engineering, Xi'an Jiaotong University, Xi'an, China; University of Ulm, Germany

## Abstract

In this paper, the synchronization problem for a class of discrete-time complex-valued neural networks with time-varying delays is investigated. Compared with the previous work, the time delay and parameters are assumed to be time-varying. By separating the real part and imaginary part, the discrete-time model of complex-valued neural networks is derived. Moreover, by using the complex-valued Lyapunov-Krasovskii functional method and linear matrix inequality as tools, sufficient conditions of the synchronization stability are obtained. In numerical simulation, examples are presented to show the effectiveness of our method.

## Introduction

In the past decades, the study of dynamical neural network has attracted researchers' attentions because of its potential applications in a variety of areas, such as image processing, combinatorial optimization problems, pattern recognition, signal processing and so on (see, for instance [1]–[5]). In the investigations of dynamical neural network, synchronization, control and stability analysis have attracted people's interests in the research of chaotic systems, complex nonlinear systems and dynamical neural networks (see [6]–[11] and references therein) since Pecora and Carroll achieved synchronization between two chaotic oscillators by PC (Pecora and Carroll) method [12]. As we know, time delays commonly exist in the neural networks because of the network traffic congestions and the finite speed of information transmission in networks. So the study of dynamic properties with time delay is of great significance and importance. However, most of the studied networks are real number valued. Currently, in order to investigate the complex properties in complex-valued neural networks, some complex-valued network models are proposed. For example, Hu gives the global stability of complex-valued recurrent continuous neural networks with time-delays [13] and Zhou further studies the boundedness and complete stability of complex-valued neural networks [14] in 2013. For discrete-time complex-valued neural networks, the boundness and stability of neural networks without time delays [15], [16] and the boundness and stability of neural networks with time delays are studied by Zhou and Duan. However, in aforementioned discrete-time complex-valued neural networks, time delays are fixed and parameters are constant, which are not suitable. It should be noticed that time delay is a common phenomenon in network because of the signal transmission. Generally, time delays can be divided into time-varying time delays and constant time delays. Time-varying time delays are different from constant delays because the delay varies with time. In practice, time delays always vary in a bounded range instead of a fixed point and the constant delays can be seen as a special case of time-varying delays. So the study of time-varying delays has more potential applications. On the other hand, the study of time-varying delays is more challenging, the derived theorems of time-varying delays can be easily applied in the network with constant delays when upper limit equals to the lower limit as 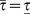. However, the corresponding theorems of constant delays are hard to be used in the study of time-varying delay network.

Motived by above discussions, by separating the real part and imaginary part of the neural networks and constructing complex Lyapunov-Krasovskii functional candidates, we will investigate the synchronization problem for a class of discrete-time complex-valued neural networks with time-varying delays. Although some sufficient conditions for stability and synchronization problems of discrete real number neural networks have been derived by some researchers, as far as we know, there has been no literatures investigate the discrete-time complex-valued neural networks with time-varying delays. We believe that the synchronization problems for such kind of networks are still remains open and challenging.

The rest paper is organized as follows. In section 2, the basic models, preliminaries and lemmas are presented. Section 3 presents stability analysis and sufficient conditions with linear matrix inequality (LMI). Some numerical simulations and examples to show the robustness and effectiveness of our methods are in section 4. Finally, we give some concluding remarks in section 5.

*Notations*: Throughout the paper, 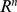 represents the *n*-dimensional Euclidean space. 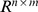 is the set of 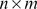 real matrices. 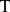 means the transpose of the corresponding matrix and the symmetric matrix 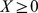 (respectively, 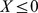,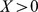 and 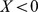) means that 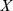 is positive semidefinite (respectively, negative semidefinite, positive definite and negative definite). 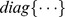 denotes a block-diagonal matrix and 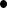 is used to represent a term induced by symmetry. If not explicitly stated, matrices dimensions are assumed to be compatible for algebraic operations.

### The system model and preliminaries

In this paper, we will consider the following discrete-time complex-valued neural network model consisting of *n* coupled nodes with time-varying delays:
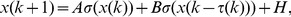
(1)where 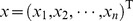
is the state vector, 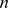
is the number of neural cells. 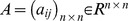, 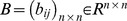 are the connection weight matrix and the delayed connection weight matrix. 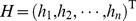 represents the input vector, 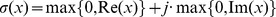 is a complex-valued function, 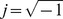. Time delay 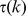 ranges from 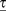 to 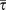 as 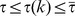. Complex-valued parameters in the neural network can be represented as 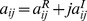, 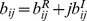. Then the model can be separated as
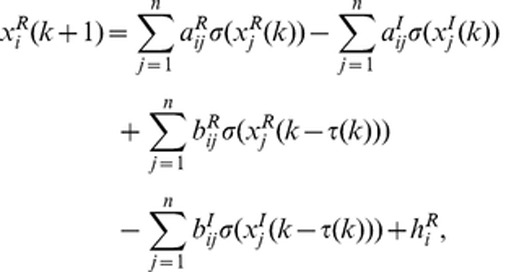
(1-1)

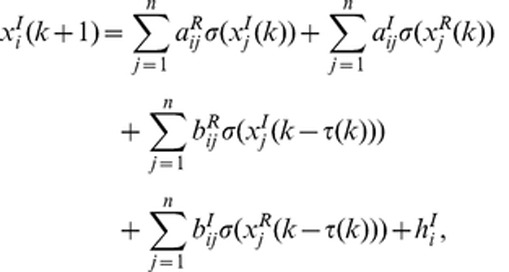
(1-2)where 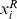 and 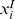 are the real part and imaginary part of variable 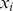, respectively. 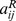 and 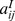 are the real part and imaginary part of connection weight 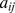 and 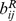 and 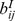 are the real part and imaginary part of delayed connection weight 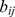. 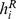 and 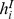 are the real part and imaginary part of input 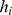. Connection weight matrices are represented as 
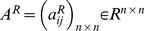
, 
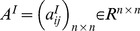
, 
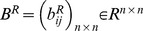
 and 
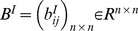
. Consider two discrete-time complex-valued neural networks with time-varying delays labeled as 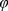 and 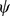. Variables at step *k* in these two neural networks are defined as 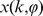 and 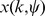 and 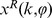, 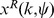, 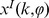 and 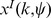 are corresponding real parts and imaginary parts. It follows from the models (1-1) and (1-2) that
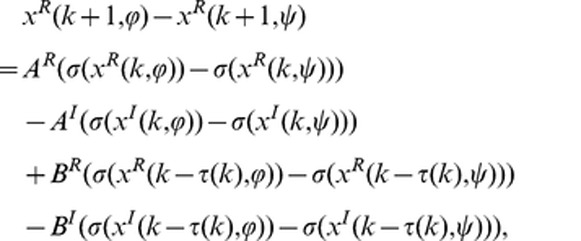
(2-1)

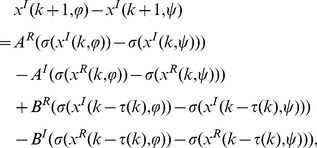
(2-2)where 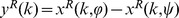, 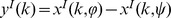, 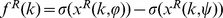, 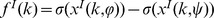. Then we have
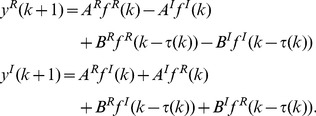
(3)


The initial condition associated with complex-valued network (3) is defined as
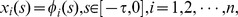
where 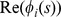 and 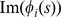 are continuous when 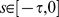.

In this paper, following definition and lemmas are listed to get the stability conditions.

**Definition** [16]:

The equilibrium point 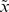 of the model (1) with the initial condition 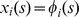 is said to be globally exponentially stable if there exist two positive constants 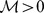 and 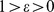 satisfies
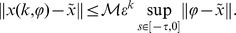



**Lemma 1** (Schur complement, [17]):

Given the following matrix
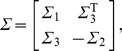
where 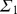
is a non-singular matrix and 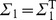, 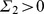 and 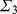 is a constant matrix, then we say 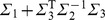 is the schur complement of 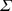 about 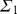 and we have the following conclusion:
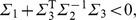
holds if and only if the following schur complement holds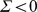.

**Lemma** 2 [18]:

Consider a symmetric positive-semidefinite matrix 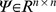 (that is to say, 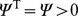), scalar 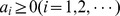 and vector 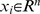. We can get the following inequality
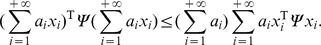



**Assumption** [13]:


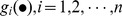
 satisfy the Lipschitz continuity condition in the complex domain, which means there exists a positive constant 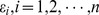 for any state variable 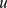 and 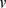, one has
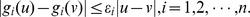



Where 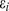 is the corresponding Lipschitz constant.

## Methods

In this section, we will deal with the synchronization problem of the aforementioned complex-valued neural networks (1). First, we will give the main result in this paper as follows.

### Theorem 1

The dynamical neural networks (1) will be globally stable if there exist positive matrices 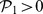, 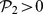, 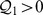 and 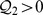, diagonal matrices 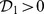, 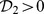, 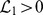, and 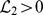, such that the following LMI holds.
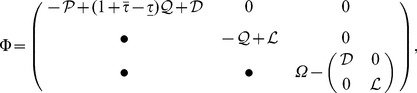
(4)where 
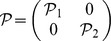
, 
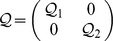
, 
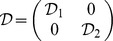
, 
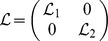
 and
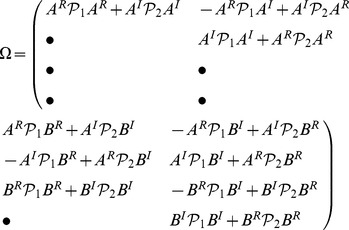



***Proof*** **: The detailed proofs for eq. (4) can be found in the Appendix S1.**

As we mentioned in the introduction, time-varying delays are completely different from constant delays. Constant delays can be seen a special case of time-varying delays when the delays vary in a fixed point. On the other hand, some existing methods can not be applied in time-varying systems. In order to show the difference, the following corollary will be presented.

### Corollary 1

For a constant time delay 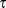, the dynamical neural networks (1) will be globally stable if there exist positive matrices 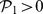, 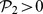, 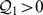,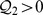, 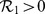 and 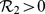, diagonal matrices 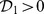, 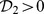, 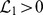, and 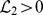, such that the following LMI holds.



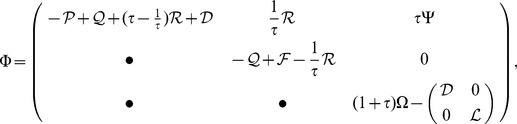
(5)where 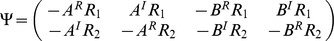 and 
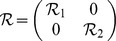
. Other variables and matrices are the same with definitions in ***theorem 1***.

***Proof*** **:** For the stability of a complex-valued neural network with constant time delay 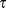, with the similar method in [16], it is easy to get the corollary.

### Remark 1

The class of complex-valued neural network with constant time delay has been investigated in [16] and theorem has been derived. Different from the previous study, the corollary 1 is established by separating the real part and imaginary part from the complex-valued neural network. Which means the problem of complex-valued neural network is translated into the stability of corresponding real neural networks and the network data are easier to handle. However, in practice, time delays are always time-varying, and then the above corollary will be infeasible.

Above linear matrix inequalities are established under the circumstance of not taking the assumption into account. If above assumption, which is an extension of the real-valued function, is satisfied. We have

### Corollary 2

The dynamical neural networks (1) will be globally stable if there exist positive matrices 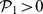, 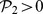, 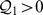 and 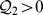, diagonal matrices 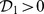, 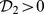, 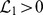, 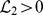, positive constants 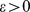, such that the following LMI holds.
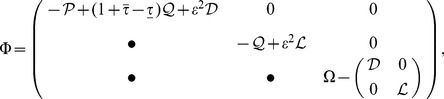
(6)where 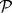, 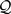, 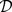, 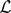 and 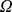 have the same meaning as in ***theorem 1***.

***Proof*** **: The detailed proofs for eq. (6) can be found in the Appendix S2.**

### Remark 2

Compare the theorem 1 with the corollary 1, we can find that the theorem is the special case when the Lipschitz constant 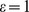. For real-valued neural network, the activation function is always chosen smooth and bounded. However, in complex-valued network, according to Liouville's theorem [19], the activation functions cannot be both bounded and analytic. Which means theorem 1 is less conservative and more general in practice.

## Results and Discussion

In this section, examples are provided to demonstrate the robustness and effective of our method.

### Example 1

Consider a two-neuron complex-valued network, where



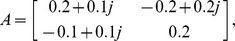


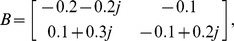


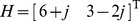



The time delays are bounded as 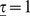 and 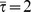, then from theorem 1, by using the LMI toolbox, we can solve the LMI (3) with the proposed matrices and parameters and the feasible results are shown as



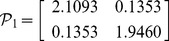


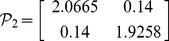





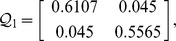


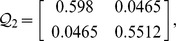





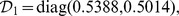


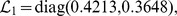





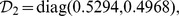


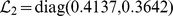



**Figure 1:**
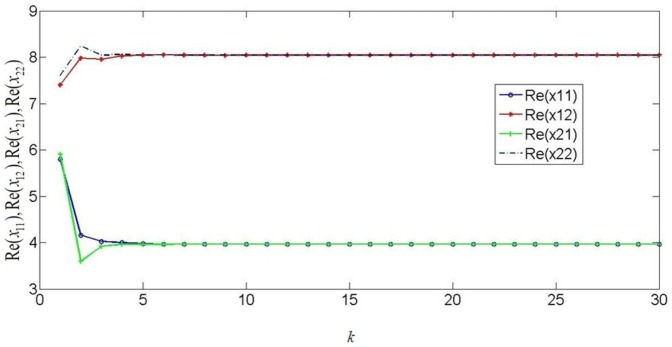
Real part of the two-neuron complex-valued neural networks.

**Figure 2:**
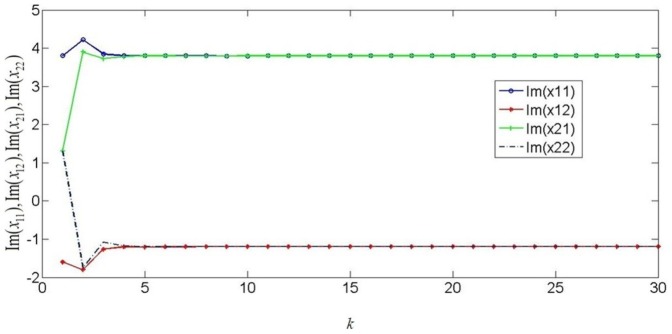
Imaginary part of the two-neuron complex-valued neural networks.

**Figure 3:**
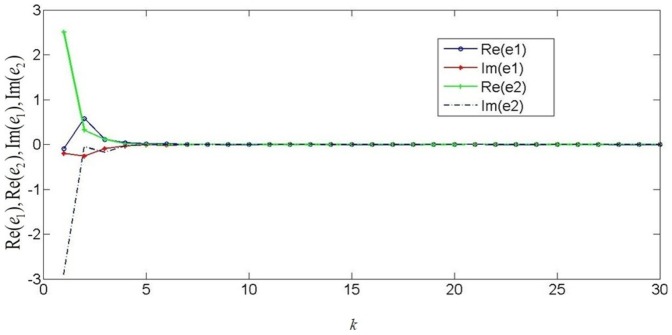
Errors of the two-neuron complex-valued neural networks.

Set the initial states as 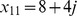,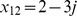, 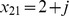 and 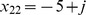. Errors are defined as 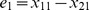 and 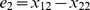. Based on the theorem 1, the discrete-time coupled neural networks with mixed time delays will get the synchronization and it is proved by numerical simulations. Fig 1 shows the real part states of discrete-time complex-valued neural networks and Fig 2 shows the imaginary part states of discrete-time complex-valued neural networks. Fig 3 depicts the errors of discrete-time complex-valued neural networks.

### Example 2

Consider a neural network with three nodes, where



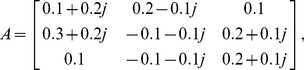


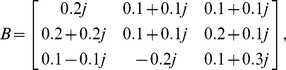





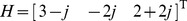



Time delays are bounded as 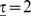 and 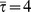, then from theorem 1 or corollary 2, by using the LMI toolbox, we can solve the LMI with the proposed matrices and parameters and the feasible results are shown as



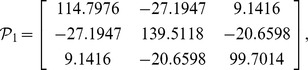


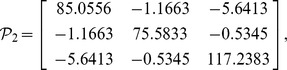





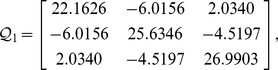


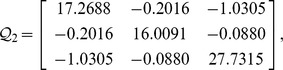





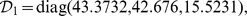


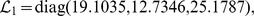





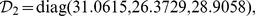


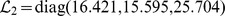
.

## Conclusion

In this article, we have presented a new approach to deal with the stability problem of discrete-time complex-valued neural networks with time-varying delays. Compared with existing results, the derived theorems and corollaries are easier to be solved with the Matlab LMI toolbox because real part and the imaginary part of complex-valued variables are separated and derived results are real number style. What's more, we propose a feasible method to solve time-varying delays of discrete-time complex-valued neural networks. Constant time delays are considered as a special case of time-varying delays in our paper and also solvable. In addition, it should be noted that time-varying delays are bounded and lager time-varying ranges will lead the LMI unsolvable. Finally, simulations show the effective and robustness of our method.

## Supporting Information

Appendix S1 **Proof of eq.(4).**
(DOC)

Appendix S2 **Proof of eq.(6).**
(DOC)
