# Supplementary material for: Stability and Synchronization for Discrete-Time Complex-Valued Neural Networks with Time-Varying Delays
Source: PLoS One. 2014 Apr 8;9(4):e93838. doi: 10.1371/journal.pone.0093838 (PMC3979734; doi:10.1371/journal.pone.0093838)
Supplement: Appendix S1 — Proof of eq.(4). (DOC) [file pone.0093838.s001.doc]

***Proof* of Eq. (4):**

In order to deal with the stability problem of the complex-valued network (1), we introduce the following Lyapunov-Krasovskii functional:

$V(k)=\sum_{i=1}^{6} V_{i}(k)$, (7)

where

, (8-1)

, (8-2)

, (9-1)

, (9-2)

, (10-1)

, (10-2)

then

, (11-1)

, (11-2)

, (12-1)

, (12-2)

, (13-1)

, (13-2)

From systems (11-1), (11-2), (12-1), (12-2), (13-1) and (13-2), one has

, (14)

where and

. (15)

From the property of $\sigma$, it is easy to get that

$\left|f^{R}(k)\right|\leq\left|y^{R}(k)\right|$, $\left|f^{I}(k)\right|\leq\left|y^{I}(k)\right|$,

and

$\left|f^{R}(k-\tau(k))\right|\leq\left|y^{R}(k-\tau(k))\right|$, $\left|f^{I}(k-\tau(k))\right|\leq\left|y^{I}(k-\tau(k))\right|$.

This means

$\left(\begin{matrix} y^{R}(k) \\ f^{R}(k) \end{matrix}\right)^{T}\left(\begin{matrix} e_{i}e_{i}^{T} & 0 \\ 0 & -e_{i}e_{i}^{T} \end{matrix}\right)\left(\begin{matrix} y^{R}(k) \\ f^{R}(k) \end{matrix}\right)\geq0$, (16-1)

$\left(\begin{matrix} y^{I}(k) \\ f^{I}(k) \end{matrix}\right)^{T}\left(\begin{matrix} e_{i}e_{i}^{T} & 0 \\ 0 & -e_{i}e_{i}^{T} \end{matrix}\right)\left(\begin{matrix} y^{I}(k) \\ f^{I}(k) \end{matrix}\right)\geq0$, (16-2)

and

$\left(\begin{matrix} y^{R}(k-\tau(k)) \\ f^{R}(k-\tau(k)) \end{matrix}\right)^{T}\left(\begin{matrix} e_{i}e_{i}^{T} & 0 \\ 0 & -e_{i}e_{i}^{T} \end{matrix}\right)\left(\begin{matrix} y^{R}(k-\tau(k)) \\ f^{R}(k-\tau(k)) \end{matrix}\right)\geq0$. (17-1)

$\left(\begin{matrix} y^{I}(k-\tau(k)) \\ f^{I}(k-\tau(k)) \end{matrix}\right)^{T}\left(\begin{matrix} e_{i}e_{i}^{T} & 0 \\ 0 & -e_{i}e_{i}^{T} \end{matrix}\right)\left(\begin{matrix} y^{I}(k-\tau(k)) \\ f^{I}(k-\tau(k)) \end{matrix}\right)\geq0$. (17-2)

For positive-semidefinite diagonal matrices , , and , since $d_{ij}\geq0$ and $l_{ij}\geq0,i=1,2,j=1,2,\cdots,n$, (16-1) follows readily that

$\sum_{j=1}^{n} d_{1j}\left(\begin{matrix} y^{R}(k) \\ f^{R}(k) \end{matrix}\right)^{T}\left(\begin{matrix} e_{i}e_{i}^{T} & 0 \\ 0 & -e_{i}e_{i}^{T} \end{matrix}\right)\left(\begin{matrix} y^{R}(k) \\ f^{R}(k) \end{matrix}\right)\geq0$, (18)

which means

. (19-1)

Similarly, from (16-2), (17-1) and (17-2) we can get that

. (19-2)

. (20-1)

. (20-2)

Therefore, we can get the following inequation from (13), (17) and (18)

$\Delta V(k)\leq\xi^{T}(k)\Phi\xi(k)$. (21)

Because $\Phi$ is negative defined, then one has

$\Delta V(k)\leq\lambda_{\max}(\Phi)\left\|\xi(k)\right\|^{2}\leq\lambda_{\max}(\Phi)\left\|y(k)\right\|^{2}$. (22)

Which implies the network is globally exponentially stable [20], this completes the proof of the theorem.
