# Supplementary material for: Stability and Synchronization for Discrete-Time Complex-Valued Neural Networks with Time-Varying Delays
Source: PLoS One. 2014 Apr 8;9(4):e93838. doi: 10.1371/journal.pone.0093838 (PMC3979734; doi:10.1371/journal.pone.0093838)
Supplement: Appendix S2 — Proof of eq.(6). (DOC) [file pone.0093838.s002.doc]

***Proof* of Eq. (6):**

Assumption implies that

,

which is equal to

$\left|f^{R}(k)\right|\leq\varepsilon\left|y^{R}(k)\right|$, $\left|f^{I}(k)\right|\leq\varepsilon\left|y^{I}(k)\right|$,

and

$\left|f^{R}(k-\tau(k))\right|\leq\varepsilon\left|y^{R}(k-\tau(k))\right|$, $\left|f^{I}(k-\tau(k))\right|\leq\varepsilon\left|y^{I}(k-\tau(k))\right|$.

From inequalities (16)-(20), one has

. (23-1)

Similarly, from (16-2), (17-1) and (17-2), we can get that

. (23-2)

. (24-1)

. (24-2)

From the theorem 1, we can easily get the corollary. That completes the proof.
